# A 120° Preoperative Knee Flexion Cutoff Identifies Patients Likely to Achieve Postoperative Flexion ≥ 120° and Clinically Meaningful Flexion Gain After Total Knee Arthroplasty

**DOI:** 10.3390/jcm15103775

**Published:** 2026-05-14

**Authors:** Mitsuhiko Kubo, Tsutomu Maeda, Kosuke Kumagai, Yasutaka Amano, Yuki Nosaka, Kazuhiro Uenaka, Hitomi Fujikawa, Sadafumi Horikawa, Taku Kawasaki, Shinji Imai

**Affiliations:** 1 Department of Sports and Musculoskeletal Pain Medicine, Shiga University of Medical Science, Otsu 520-2192, Japan; 2 Department of Orthopaedic Surgery, Shiga University of Medical Science, Otsu 520-2192, Japan; tsmaeda@belle.shiga-med.ac.jp (T.M.); kumamp@belle.shiga-med.ac.jp (K.K.); amano021@belle.shiga-med.ac.jp (Y.A.); sukeki@belle.shiga-med.ac.jp (Y.N.); kaazuhi@belle.shiga-med.ac.jp (K.U.); hitomio917@yahoo.co.jp (H.F.); sadafumi3101@gmail.com (S.H.); tka@belle.shiga-med.ac.jp (T.K.); simai@belle.shiga-med.ac.jp (S.I.)

**Keywords:** total knee arthroplasty, range of motion, patient-reported outcome measure, patient satisfaction, knee function

## Abstract

**Background/Objectives:** In our previous study, we reported that postoperative flexion ≥ 120° is associated with better knee function after TKA, whereas flexion gain is associated with higher patient satisfaction. However, preoperative determinants of achieving these clinically relevant targets remain unclear. This study investigated preoperative factors predicting (1) postoperative flexion ≥ 120° and (2) clinically meaningful flexion gain after TKA. **Methods:** We retrospectively reviewed prospectively collected data from 221 primary TKAs (171 patients) performed between 2014 and 2020. Passive knee range of motion (ROM) was measured preoperatively and at 1 year postoperatively. Preoperative variables included age, sex, body mass index (BMI), Pain Catastrophizing Scale, Knee Society Score, and Knee Injury and Osteoarthritis Outcome Score (KOOS). Patients were categorized as the good-flexion group (postoperative flexion ≥ 120°) or poor-flexion group (<120°), and as the improvement group (flexion gain ≥ 5°) or no-improvement group (<5°). Variables differing between groups were entered into multivariable logistic regression. Receiver operating characteristic (ROC) analysis (Youden index) identified optimal cutoffs. **Results:** Postoperative flexion was ≥120° in 63.3% of knees, and flexion gain ≥ 5° occurred in 48.4%. In multivariable models, preoperative flexion angle was the only independent preoperative predictor of achieving postoperative flexion ≥ 120° (*p* < 0.001) and flexion gain ≥ 5° (*p* < 0.001). ROC analysis showed that a preoperative flexion cutoff of 120° best discriminated both outcomes (AUC 0.78 for postoperative flexion ≥ 120°; AUC 0.80 for flexion gain ≥ 5°). **Conclusions:** A 120° preoperative knee flexion threshold provides a simple, clinically actionable marker for predicting postoperative flexion ≥ 120° and meaningful flexion gain after TKA. Incorporating preoperative flexion into shared decision-making may improve counseling by setting realistic expectations for postoperative knee function and satisfaction.

## 1. Introduction

Total knee arthroplasty (TKA) is an effective treatment for end-stage knee joint disorders and typically provides substantial pain relief. However, functional recovery after TKA varies considerably among patients, and a meaningful proportion remain dissatisfied with the surgical outcome. In recent years, patient-reported outcome measures (PROMs) and patient satisfaction have therefore become key endpoints for evaluating TKA. Nevertheless, reported satisfaction rates remain approximately 80% [1,2], indicating room for improvement. Range of motion (ROM) is a traditional and clinically intuitive outcome measure after TKA because it is easy for both patients and clinicians to understand and assess. In our previous work, we examined the relationships among ROM, PROMs, and patient satisfaction, and found that postoperative knee flexion of ≥120° is associated with better knee function, whereas improvement in flexion angle is associated with higher patient satisfaction after TKA. Being able to predict postoperative function and satisfaction before surgery would help optimize surgical indications and facilitate shared decision-making and patient counseling. However, preoperative determinants of achieving adequate postoperative flexion and improvement in flexion remain incompletely understood and are likely multifactorial. We therefore conducted this study to identify preoperative factors associated with (i) achieving postoperative knee flexion of ≥120° and (ii) improvement in flexion angle at 1 year after TKA.

Accordingly, the purpose of this study was to investigate preoperative predictors of postoperative flexion of ≥120° and improvement in knee flexion after TKA. We measured ROM preoperatively and at 1 year postoperatively and assessed preoperative patient-related factors (age, sex, body mass index [BMI], and pain catastrophizing assessed using the Pain Catastrophizing Scale [PCS]) and clinical outcomes (ROM, Knee Society Score, and Knee Injury and Osteoarthritis Outcome Score). We then compared these preoperative factors between patients with postoperative flexion ≥ 120° versus <120°, and between those with improved flexion versus no improvement at 1 year. Finally, we explored potential cutoff values for the identified preoperative predictors.

In our prior cohort study, we identified postoperative flexion ≥ 120° as a practical target associated with better knee function and a flexion gain ≥ 5° as a target associated with higher patient satisfaction after TKA [3]. The present study extends these findings by examining which readily available preoperative factors predict whether patients will achieve these clinically relevant targets at 1 year after TKA, thereby supporting preoperative counseling and shared decision-making. Therefore, in the present study, we prespecified postoperative flexion ≥ 120° and flexion gain ≥ 5° as clinically relevant targets based on our prior work, rather than optimizing these thresholds post hoc in the current dataset.

## 2. Materials and Methods

We retrospectively reviewed the prospectively collected data of 171 patients (143 female, 28 male) who underwent 221 primary TKA procedures (184 female knees, 37 male knees) between 19 June 2014 and 10 December 2020. The average patient age was 72.8 years (range, 33–89 years) and the mean BMI was 25.9 kg/m^2^ (range, 16.0–40.8 kg/m^2^) at the time of the surgery. The study population included 195 knees with osteoarthritis, 20 knees with rheumatoid arthritis, and 6 knees with osteonecrosis. The study protocol was approved by the institutional review board of the authors’ affiliated institution.

The ROM (passive extension and flexion) was measured to the nearest 5° by using a goniometer while the patient was in the supine position, and measurements were obtained preoperatively and at the 1-year postoperative follow-up visit. Flexion contracture was expressed as a negative value. Preoperatively, we evaluated catastrophic thinking using the Pain Catastrophizing Scale (PCS) while knee function was assessed using the Knee Society Score (KSS) and the Knee Injury Osteoarthritis Outcome Score (KOOS). The PCS is a self-administered questionnaire used to detect catastrophic thinking, with scores ranging from 0 to 52. The KSS is a conventional doctor-derived assessment consisting of two components, the knee score/functional score, each with a maximum score of 100. The KOOS is based on five subscales (symptoms/pain/function in daily living [ADL]/function in sport and recreation function [Sports/Rec]/knee-related quality of life [QOL]), with scores standardized to a scale from 0 (worst) to 100 (best).

Patients were classified into two groups based on our previously reported criteria of a postoperative flexion angle of 120° for good knee function and a flexion angle improvement of 5° for good postoperative patient satisfaction. Patients with a postoperative flexion angle of 120° or more were defined as the good flexion group, and those with a postoperative flexion angle of less than 120° were defined as the poor flexion group. Patients showing an improvement of 5° or more in the flexion angle were defined as the improvement group, and those showing an improvement of less than 5° in the flexion angle were defined as the no-improvement group. These cutoffs (postoperative flexion ≥ 120° and flexion gain ≥ 5°) were prespecified a priori based on our prior report [3] and were not data-driven thresholds derived from the current cohort. The subsequent statistical analyses were performed for both comparisons: good flexion vs. poor flexion and improvement vs. no improvement.

### Statistical Analysis

We compared patient factors (age, BMI, catastrophic thinking), clinical outcomes (KSS, KOOS), and preoperative ROM (extension and flexion) between the good- and poor-flexion groups, as well as between the improvement and the no-improvement groups by using the Mann–Whitney U test. Sex was also compared between the two groups by using the chi-squared test. Multivariable logistic regression analysis was performed using variables that differed between groups in univariable analyses to avoid overfitting given the sample size. In addition, receiver operating characteristic (ROC) curve analysis was performed on the significant variables obtained from the multivariable logistic regression analysis, and the cutoff points at which 120° of postoperative flexion was obtained and the postoperative flexion angle was improved were determined using Youden’s index. EXCEL Toukeikaiseki, EXCEL Tahenryoukaiseki Ver.8.0 (Esumi, Tokyo, Japan) was used to conduct statistical analyses, and a value of *p* < 0.05 was considered significant. 

Sensitivity analysis: Because some patients underwent bilateral TKA and the primary dataset included 221 knees from 171 patients, we performed a sensitivity analysis restricted to one knee per patient (n = 171) using the same multivariable logistic regression models with the same covariates to minimize potential within-patient correlation; results were compared with the primary analysis to assess consistency.

## 3. Results

The extension angle improved significantly from a mean (range) of −9.6° (−30° to 0°) before surgery to a mean (range) of −2.3° (−20° to 0°) after surgery (*p* < 0.01). The flexion angle improved significantly from a mean (range) of 116.3° (70° to 150°) before surgery to a mean (range) of 119.8° (65° to 150°) after surgery (*p* = 0.009). The preoperative findings were as follows: mean PCS score was 32.1 (0–52), mean KSS knee score was 38.9 (0–77), mean KSS function score was 42.5 (0–90), mean KOOS symptom score was 54.3 (3.6–100), mean KOOS pain score was 44.5 (2.8–94.4), mean KOOS ADL score was 54.7 (10.3–98.5), mean KOOS Sports/Rec score was 16.5 (0–70), and mean KOOS QOL score was 5.4 (0–87.5) (Table 1). The good-flexion group consisted of 140 patients (63.3%) and the poor-flexion group consisted of 81 patients (36.7%). Patient factors, clinical evaluation, and preoperative ROM for the good-flexion group and poor-flexion group are shown in Table 2, and the two groups showed significant differences in BMI (25.1 vs. 27.2, *p* < 0.001), KSS knee score (40.7 vs. 35.9, *p* = 0.029), KOOS Sports/Rec score (18.3 vs. 13.2, *p* = 0.02), preoperative extension angle (−8.6 vs. −11.3, *p* = 0.01) and flexion angle (121.7 vs. 106.6, *p* < 0.001). In the multivariable logistic regression analysis, where postoperative flexion was used as the objective variable, and BMI, KSS knee score, KOOS Sports/Rec score, preoperative extension, and flexion angle were used as explanatory variables, only the preoperative flexion angle had a significant effect (*p* < 0.001). The improvement group consisted of 107 patients (48.4%), and the no-improvement group consisted of 114 patients (51.6%). Patient factors, clinical evaluation, and preoperative ROM for the improvement and no-improvement groups are shown in Table 3; significant differences were observed in the KSS knee score (35.6 vs. 42.0, *p* = 0.003), preoperative extension (−10.7° vs. −8.5°, *p* = 0.03), and flexion angle (108.2° vs. 123.5°, *p* < 0.001). In the multivariable logistic regression analysis, where the improvement in flexion angle was used as the objective variable, and the KSS knee score, preoperative extension, and flexion angle were used as explanatory variables, only the preoperative flexion angle had a significant effect (*p* < 0.001). 

The area under the curve (AUC) for the postoperative flexion angle with the preoperative flexion angle determined by ROC curve analysis was 0.78. The cutoff preoperative flexion angle for a postoperative flexion angle greater than 120° was 120° (Table 4). The AUC for the improvement in flexion angle with the preoperative flexion angle determined by ROC curve analysis was 0.80. The cutoff preoperative flexion angle for an improvement of >5° in the flexion angle was also 120° (Table 4). In a sensitivity analysis restricted to one knee per patient (n = 171), preoperative flexion angle remained the only independent predictor of postoperative flexion ≥ 120° and flexion gain ≥ 5°, consistent with the primary analysis (Table S1).

## 4. Discussion

We had previously investigated the relationships among ROM, postoperative knee function, and patient satisfaction after TKA. Our results suggested that a postoperative flexion of 120° is necessary to obtain good knee function, and that an improvement of 5° in the flexion angle is necessary to obtain good patient satisfaction [3]. The purpose of this study was to investigate the preoperative factors that lead to a postoperative flexion of 120° and an improved flexion angle, that is, good knee joint function and patient satisfaction after TKA. The preoperative factor influencing a postoperative flexion of 120°, that is, good postoperative knee joint function, was a preoperative flexion angle of ≥120°. On the other hand, the preoperative factor that influenced the achievement of an improved flexion angle, that is, good postoperative patient satisfaction, was a preoperative flexion angle of ≤120°.

The only preoperative factor that was significantly different between patients with postoperative flexion angles of ≥120° and <120° was the preoperative flexion angle, and a preoperative flexion angle of 120° was the cutoff value for a postoperative flexion angle of 120° or more. In other words, the only preoperative factor for obtaining postoperative flexion of 120° was a preoperative flexion angle of ≥120°. The influence of the preoperative flexion angle on the postoperative flexion angle has long been reported. As early as 1979, Ritter et al. stated that ‘the amount of flexion to be achieved postoperatively may be determined by the amount of flexion the patient had preoperatively’ [4]. Since then, the relationships between the postoperative flexion angle and patient demographics (age, sex, BMI, previous surgery, preoperative ROM, quadriceps strength, preoperative KSS, patellofemoral function), radiographic measurements (preoperative tibiofemoral alignment, joint line change, shift and tilt and height of patella, change in leg length, anteroposterior placement of the tibial component), and operative factors (component type, soft tissue release, and osteophyte removal), have been investigated, and many reports have indicated that the preoperative flexion angle is the most influential factor [5,6,7,8,9]. Gatha et al. investigated the relationship of the postoperative flexion angle with patient sex, age, preoperative flexion angle, preoperative KSS, and radiographic parameters (alignment, patellar thickness, patellar height), and, similar to our study, they reported that only preoperative flexion was significantly correlated with the postoperative flexion angle [10]. Previous studies investigated the effects of sex, age, BMI, surgical history, preoperative flexion angle, preoperative extension angle, preoperative flexion arc, and preoperative tibiofemoral angle on the postoperative flexion angle and concluded that the preoperative flexion angle had the greatest effect. In their results, an increase of 10° in the preoperative flexion angle was associated with an increase of 6.4° in the postoperative flexion angle [11]. To the best of our knowledge, there is only one report has stated that the preoperative flexion angle does not affect the postoperative flexion angle. Russell et al. compared the postoperative flexion angle between patients with preoperative flexion angles of <95° and >95°, and concluded that the postoperative flexion angle did not change in relation to the preoperative flexion [12]. Thus, most previous reports have clearly identified the preoperative flexion angle as a factor affecting the postoperative flexion angle, and our results are consistent with these reports.

Nevertheless, only a few studies have actually investigated the relationship between the preoperative flexion angle and postoperative knee function [13,14]. Khatri et al. compared postoperative KSS between patients with preoperative flexion angles of less than 50° and greater than 90° and concluded that KSS in patients with a preoperative ROM < 50° was significantly lower than that in patients with ROM > 90° [13]. Moreover, Winemaker et al. compared postoperative KSSs and Oxford Knee Scores (OKSs) between patients with preoperative flexion angles of <80° and >100° and concluded that KSSs and OKSs were significantly inferior in the stiff knee group [14]. In other words, poor preoperative flexion angles are associated with poor postoperative knee function. Combining the results of the current study with our previous report, we can conclude that a preoperative flexion angle of ≥120° results in a postoperative flexion angle of ≥120°, which in turn results in good knee function.

In addition, the only preoperative factor that was significantly different between patients with and without an improvement in flexion angle was only the preoperative flexion angle, and the cutoff value for an improvement in flexion angle was a preoperative flexion angle of 120°. Anouchi et al. classified patients into three groups according to preoperative flexion angle: those with preoperative flexion angle less than 90°, those with preoperative flexion angle 91–105°, and those with a preoperative flexion angle more than 105°. They concluded that those with low preoperative flexion angles increased their flexion postoperatively, those with midrange preoperative motion remained in the same range postoperatively, and those with a high preoperative flexion angle lost motion [15]. Moreover, Harvey et al. reported a significant negative correlation between the preoperative flexion angle and the amount of improvement in the flexion angle, and concluded that the stiffer knees gained flexion while the more mobile knees lost some of it [16]. Regarding specific preoperative flexion angles, previous reports have suggested that patients with preoperative flexion angles of 95° [4], 105° [17], and 110° [18] or more do not improve their flexion angles. A recent report suggested that the postoperative flexion angle can be improved even in patients with a preoperative flexion angle of 110–130° [19], and the cutoff preoperative value for obtaining a postoperative improvement appears to be gradually changing with improvements in implants and surgical techniques. Schurman et al. examined the effect of 13 preoperative factors and 4 postoperative factors on the postoperative flexion angle and concluded that the most influential factor was the preoperative flexion angle. However, since the effect was only about half of the total, they also showed the limitations of predicting the postoperative flexion angle on the basis of the preoperative flexion angle alone [7]. Our finding showing that the postoperative flexion angle improved in patients with preoperative flexion angles of ≤120° is consistent with recent reports.

Only a few previous reports have described the effect of the preoperative flexion angle on patient satisfaction. Kim and Jacobs compared the preoperative flexion angle between the satisfied and unsatisfied groups after TKA surgery and reported that the unsatisfied group had a significantly better preoperative flexion angle [20,21]. Patients with better knee flexion preoperatively were more likely to be dissatisfied postoperatively, with an odds ratio of 1.008 [22]. The odds ratio of patients who were unsatisfied after TKA surgery was reported to be 0.55 if the preoperative flexion is less than 90° [1]. All of these reports show that the worse the preoperative flexion angle, the better the patient satisfaction.

These finding also support the combined conclusions of this study and our previous reports. In other words, if the preoperative flexion angle is less than 120°, the flexion angle will be improved postoperatively and the patient will be more satisfied.

In summary, the preoperative flexion angle should be ≥120° in order to achieve good postoperative knee function, and the preoperative flexion angle should be ≤120° to achieve good postoperative patient satisfaction. Therefore, a preoperative flexion angle around 120° may serve as a practical reference point for preoperative counseling, expectation management, and functional goal setting in patients undergoing TKA. At present, there are no obvious indications for TKA, and patients with pain that does not respond to conservative therapy and marked degeneration (Ahlbäck grade II or Kellgren–Lawrence grade III or higher) are indicated [23,24]. In this study, postoperative knee function and the likelihood of flexion improvement could be estimated using preoperative flexion angle, which may help support shared decision-making and patient counseling when discussing expected functional outcomes after TKA. Importantly, due to the retrospective observational design, these findings should be interpreted as associations and do not establish causality. Future prospective studies are warranted to confirm whether achieving these ROM targets translates into improved patient-reported outcomes.

## 5. Limitations

The present study had some limitations. First, the sample size was relatively small. Second, this was a retrospective study. Third, the conditions used in this study, i.e., a postoperative flexion angle of 120° for good postoperative knee function and an improvement in the flexion angle to ensure good patient satisfaction, are based our previous research. However, because the preoperative ROM of patients who undergo TKA and the postoperative ROM gained after TKA depends on the hospital, it is not always possible to apply this condition. However, our previous study was examined on 500 cases, and this condition was generally indicated.

## 6. Conclusions

A 120° preoperative knee flexion threshold provides a simple, clinically actionable marker for predicting postoperative flexion ≥120° and meaningful flexion gain after TKA. Incorporating preoperative flexion into shared decision-making may improve counseling by setting realistic expectations for postoperative knee function and satisfaction. These findings reflect associations and do not establish causality.

## Figures and Tables

**Table 1:** Patient demographic characteristics.

Demographic Characteristics	Results
Sex (female/male)	184/37
Age (years)	72.8 (33–89)
BMI (kg/m^2^)	25.9 (16.0–40.8)
PCS (points)	32.3 (0–52)
KSS knee (points)	38.9 (0–77)
KSS function (points)	42.3 (0–90)
KOOS Symptoms (points)	54.5 (3.6–100)
KOOS Pain (points)	44.7 (2.8–94.4)
KOOS ADL (points)	54.7 (10.3–98.5)
KOOS Sports/Rec (points)	16.4 (0–70)
KOOS QOL (points)	25.4 (0–87.5)
Preoperative extension angle (°)	−9.6 (−30–0)
Preoperative flexion angle (°)	116.1 (70–160)

**Table 2:** Comparison between the good flexion and poor flexion groups.

	The Good Flexion Group	The Poor Flexion Group	*p*-Value
Sex (female/male)	118/22	66/15	0.25
Age (years)	73.5	71.6	0.08
BMI (kg/m^2^)	25.1	27.2	<0.001
PCS (points)	32.9	31.1	0.27
KSS knee (points)	40.7	35.9	0.03
KSS function (points)	43.1	40.9	0.46
KOOS Symptoms (points)	55.3	53.1	0.43
KOOS Pain (points)	44.8	44.5	0.90
KOOS ADL (points)	55.1	54.0	0.64
KOOS Sports/Rec (points)	18.3	13.2	0.02
KOOS QOL (points)	26.0	24.5	0.49
Preoperative extension angle (°)	−8.6	−11.3	0.01
Preoperative flexion angle (°)	121.7	106.6	<0.001

**Table 3:** Comparison between the improvement and non-improvement groups.

	The Improvement Group	The No-Improvement Group	*p*-Value
Sex (female/male)	88/19	97/17	0.15
Age (years)	72.7	72.9	0.89
BMI (kg/m^2^)	25.9	25.8	0.87
PCS (points)	32.1	32.4	0.85
KSS knee (points)	35.6	42.0	0.003
KSS function (points)	39.9	44.5	0.1
KOOS Symptoms (points)	53.7	55.2	0.59
KOOS Pain (points)	54.6	45.7	0.43
KOOS ADL (points)	54.7	54.6	0.96
KOOS Sports/Rec (points)	15.6	17.2	0.43
KOOS QOL (points)	24.1	26.6	0.92
Preoperative extension angle (°)	−10.7	−8.5	0.03
Preoperative flexion angle (°)	108.2	123.5	<0.001

**Table 4:** Receiver operating characteristic curve analysis.

	Parameter	Cut-Off Point	Odds Ratio	95% CI	*p*-Value	AUC
The poor/good flexion group	Preoperative flexion	120°	5.6	0.72–0.84	<0.001	0.78
The improvement/ no-improvement group	Preoperative flexion	120°	10.6	0.75–0.86	<0.001	0.80

## Data Availability

The original contributions presented in this study are included in the article. Further inquiries can be directed to the corresponding author.

## Supplementary Materials

The following supporting information can be downloaded at https://www.mdpi.com/article/10.3390/jcm15103775/s1, Table S1: Sensitivity analysis using one knee per patient (n = 171)—multivariable logistic regression for postoperative flexion ≥ 120° and flexion gain ≥ 5° after total knee arthroplasty.
