# Supplementary material for: A 120° Preoperative Knee Flexion Cutoff Identifies Patients Likely to Achieve Postoperative Flexion ≥ 120° and Clinically Meaningful Flexion Gain After Total Knee Arthroplasty
Source: J Clin Med. 2026 May 14;15(10):3775. doi: 10.3390/jcm15103775 (PMC13207042; doi:10.3390/jcm15103775)
Supplement: Supplementary file 1 [file jcm-15-03775-s001.zip › jcm-4280410-supplementary.pdf]

Supplementary Materials:

The following supporting information can be downloaded as part of this paper:

Table S1: Sensitivity analysis using one knee per patient (n = 171)—multivariable logistic regression for postoperative flexion ≥120° and flexion gain ≥5° after total knee arthroplasty.

Predictor	Postop flexion≥120° Adjusted OR (95% CI)	P-value	Flexion improvement ≥5° Adjusted OR (95% CI)	P-value
BMI	0.94 (0.86–1.02)	0.141	—	—
KOOS SP	1.11 (0.97–1.27)	0.131	—	—
KSS knee score	1.00 (0.97–1.02)	0.855	0.99 (0.97–1.02)	0.487
Preop extension	1.01 (0.96–1.06)	0.704	1.00 (0.95–1.05)	0.970
Preop flexion	1.09 (1.06–1.13)	<0.001	0.92 (0.89–0.95)	<0.001

Table note (for Supplementary Materials): Because the main analysis included 221 knees from 171 patients (including bilateral procedures), this sensitivity analysis was conducted using one knee per patient (n = 171) to minimize within-patient correlation. Models were constructed using the same covariates as in the main analysis; variables not included in a given model are shown as "—". Preoperative flexion angle remained the only significant independent predictor of both achieving postoperative flexion ≥120° and flexion gain ≥5°.

Abbreviations: BMI, body mass index; CI, confidence interval; KOOS, Knee injury and Osteoarthritis Outcome Score; Sports/Rec, function in sport and recreation subscale of KOOS; KSS, Knee Society Score; OR, odds ratio; TKA, total knee arthroplasty.
